# Integrative radiomics clustering analysis to decipher breast cancer heterogeneity and prognostic indicators through multiparametric MRI

**DOI:** 10.1038/s41523-024-00678-8

**Published:** 2024-08-07

**Authors:** Yongsheng He, Shaofeng Duan, Wuling Wang, Hongkai Yang, Shuya Pan, Weiqun Cheng, Liang Xia, Xuan Qi

**Affiliations:** 1 Department of Radiology, Ma’anshan People’s Hospital, Ma’anshan, Anhui 243000 China; 2 Ma’anshan Clinical College, Anhui Medical University, Hefei, Anhui 230032 China; 3 Department of Radiology, Sir Run Run Hospital affiliated to Nanjing Medical University, Nanjing, Jiangsu 211000 China

**Keywords:** Breast cancer, Breast cancer

## Abstract

Breast cancer diagnosis and treatment have been revolutionized by multiparametric Magnetic Resonance Imaging (mpMRI), encompassing T2-weighted imaging (T2WI), Diffusion-weighted imaging (DWI), and Dynamic Contrast-Enhanced MRI (DCE-MRI). We conducted a retrospective analysis of mpMRI data from 194 breast cancer patients (September 2019 to October 2023). Using ‘pyradiomics’ for radiomics feature extraction and MOVICS for unsupervised clustering. Interestingly, we identified two distinct patient clusters associated with significant differences in molecular subtypes, particularly in Luminal A subtype distribution (*p* = 0.03), estrogen receptor (ER) (*p* = 0.01), progesterone receptor (PR) (*p* = 0.04), mean tumor size (*p* < 0.01), lymph node metastasis (LNM) (*p* = 0.01), and edema (*p* < 0.01). Our study emphasizes mpMRI’s potential in breast cancer by using radiomics-based cluster analysis to categorize tumors, uncovering heterogeneity, and aiding in personalized treatment strategies.

## Introduction

Breast cancer, a leading cause of cancer-related mortality among women worldwide, presents significant diagnostic challenges due to its heterogeneity^1^. Recent advancements in multiparametric MRI have marked a significant leap in breast cancer diagnostics. This technique combines various imaging sequences to offer a comprehensive assessment of tumor characteristics, even capturing the complex heterogeneity within breast tumors^2^. Radiomics, an emerging field, further augments the potential of multiparametric MRI. It involves extracting a large number of quantitative features from medical images, uncovering patterns that may not be visible to the human eye^3^. These features encompass a wide array of data points, including shape, intensity, texture, and wavelet features, each offering insights into the tumor’s phenotype and underlying biology. The power of radiomics lies in its ability to transform static images into mineable data, opening new avenues for understanding tumor characteristics and their clinical implication.

Recent advancements in multiparametric MRI have marked a significant leap in breast cancer diagnostics. This technique, which combines various imaging sequences, offers a comprehensive assessment of tumor characteristics. Each sequence in multiparametric MRI contributes uniquely to the overall picture: T2-weighted imaging provides detailed anatomical information, diffusion-weighted imaging offers insights into cellular density, and dynamic contrast-enhanced sequences shed light on vascular properties of the tumor^4^. Many studies underscore the potential of multiparametric MRI and radiomics in enhancing our understanding of breast cancer. However, they also highlight the complexity of this approach^5^. The sheer volume and variety of data generated by radiomics analysis require sophisticated algorithms and machine-learning techniques for interpretation. Lee et al.‘s work on using machine learning for radiomic analysis is a testament to this, where they employed various algorithms to correlate MRI parameters with prognostic biomarkers and molecular subtypes of breast cancer^6^. The integration of these advanced imaging and analytical techniques represents a significant stride towards personalized medicine in breast cancer care^7^. It allows for a more nuanced understanding of the disease, potentially leading to more tailored and effective treatment strategies. However, the field is still evolving, and ongoing research is crucial to fully harness the potential of these technologies.

Despite these advancements, there remains a notable research gap in the specific application of radiomics clustering analysis in breast cancer using advanced MRI technologies^8^. Clustering, an unsupervised learning technique, has effectively been applied to identify inherent groupings in patient samples, utilizing a specific similarity measure for visualization and partitioning purposes, the application value in the analysis of histopathological characteristics and prognosis of stage I lung adenocarcinoma has been reported^9^. This study aims to bridge this gap by evaluating the application value of multiparametric imaging radiomics clustering analysis in breast cancer, utilizing the advanced capabilities of the 3.0 T MRI scanner. The study focuses on a detailed analysis of radiomics features extracted from multiparametric MRI scans, correlating these features with histopathological data to enhance the accuracy and specificity of breast cancer classification. By doing so, the study endeavors to contribute to the nuanced understanding of breast cancer phenotypes and to aid in the development of more personalized and effective treatment strategies.

## Results

### Patient and tumor characteristics

A total of 194 women were included in the study, with an average age of 54.9 ± 10.8 years. The key findings include a predominance of Luminal B subtype at 47.7% (92/194), followed by Luminal A at 22.3% (43/194). Hormone receptor analysis showed that 71.0% (137/194) of the patients were ER-positive and 49.2% (95/194) were PR-positive, with 58.5% (113/194) exhibiting HER2 positivity. Ki67 expression was high in 64.2% (124/194) of patients. The tumor size was 2.51 ± 1.53 cm, and volume was 4336.4 ± 5175.4 mm^3^. Histologically, most tumors were Grade II, at 60.6% (117/194). LNM was present in 41.5% (80/194) of cases, and lymphovascular invasion in 25.9% (50/194). Fibroglandular tissue distribution varied, with the majority in categories c (51.8%, 100/194) and b (31.1%, 60/194). BPE was mostly minimal (37.3%, 72/194) or mild (50.8%, 98/194). The majority of tumors were mass type (91.2%, 176/194), with TIC Type III being the most common (58.0%, 112/194). Edema was present in 50.8% (98/194) of cases, and DWI signals were predominantly high (92.7%, 179/194) (Table 1). These comprehensive results provide a detailed overview of the clinical and pathological characteristics of the breast cancer cases in this study.

**Table 1:** Clinical, Histologic, and Radiological characteristics of patients

Characteristic	Level	Finding (*n* = 194)
Age (years)*	Mean ± SD	54.9 ± 10.8
Molecular subtypes	Luminal A	43 (22.3)
	Luminal B	92 (47.7)
	HER2-positive	18 (9.3)
	Basal-like	40 (20.7)
ER	Negative	56 (29.0)
	Positive	137 (71.0)
PR	Negative	98 (50.8)
	Positive	95 (49.2)
HER2	Negative	80 (41.5)
	Positive	113 (58.5)
Ki67	<14%	69 (35.8)
	≥14%	124 (64.2)
Maximum (cm)*	Mean ± SD	2.5 ± 1.5
Volume (mm^3^)	Mean ± SD	4336.4 ± 5175.4
Histological grades	I	55 (28.5)
	II	117 (60.6)
	III	21 (10.9)
LNM	Negative	113 (58.5)
	Positive	80 (41.5)
LVI	Negative	143 (74.1)
	Positive	50 (25.9)
Around	Left	93 (48.2)
	Right	100 (51.8)
FGT	a	9 (4.7)
	b	60 (31.1)
	c	100 (51.8)
	d	24 (12.4)
Location	Superior external	87 (45.1)
	Inferior external	28 (14.5)
	Superior internal	40 (20.7)
	Inferior internal	16 (8.3)
	Posterior areola	14 (7.3)
	Central region	8 (4.1)
BEP	Minimal	72 (37.3)
	Mild	98 (50.8)
	Moderate	20 (10.4)
	Significant	3 (1.6)
Type	Non-mass	17 (8.8)
	Mass	176 (91.2)
TIC	I	7 (3.6)
	II	74 (38.3)
	III	112 (58.0)
Edema	No	95 (49.2)
	Yes	98 (50.8)
T2WI signals	Low	50 (25.9)
	Equal	72 (37.3)
	High	71 (36.8)
DWI signals	Low	1 (0.5)
	Equal	13 (6.7)
	High	179 (92.7)

*ER* estrogen receptor, *PR* progesterone receptor, *LNM* lymph node metastasis, *LVI* lymphovascular invasion, *FGT* fibro glandular tissue, *BEP* background parenchymal Enhancement, *TIC* time signal intensity curve, *SD* standard deviation. Unless otherwise indicated, data are numbers of patients and data in parentheses are percentages. * Data in parentheses are mean ± SD.

### Clustering analysis

**Fig. 1:**
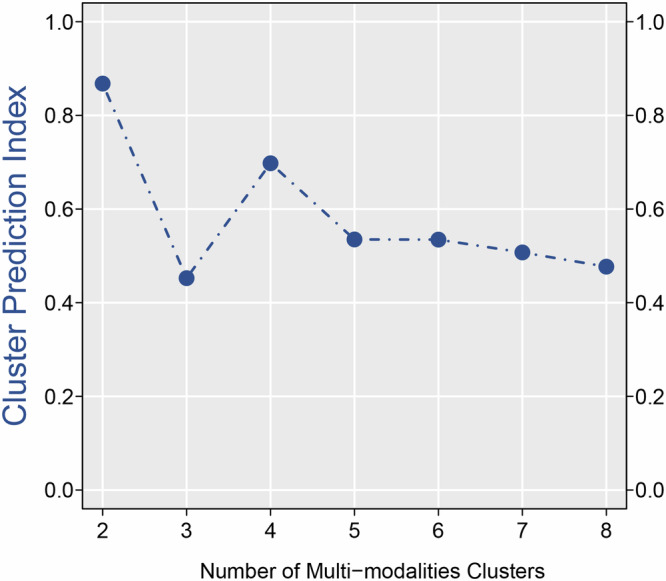
Utilizing the CPI to determine the optimal k by calculating it across a range of k values from 2 to 8. The CPI assesses each potential clustering solution by considering factors such as within-cluster similarity and between-cluster differences, aiming to identify the clustering configuration that offers the most distinct and cohesive groups. The optimal k was identified as the one with the maximum CPI value. The data indicates that the CPI reaches its peak at k = 2, suggesting that a classification count of 2 is the most optimal for this analysis.

**Fig. 2:**
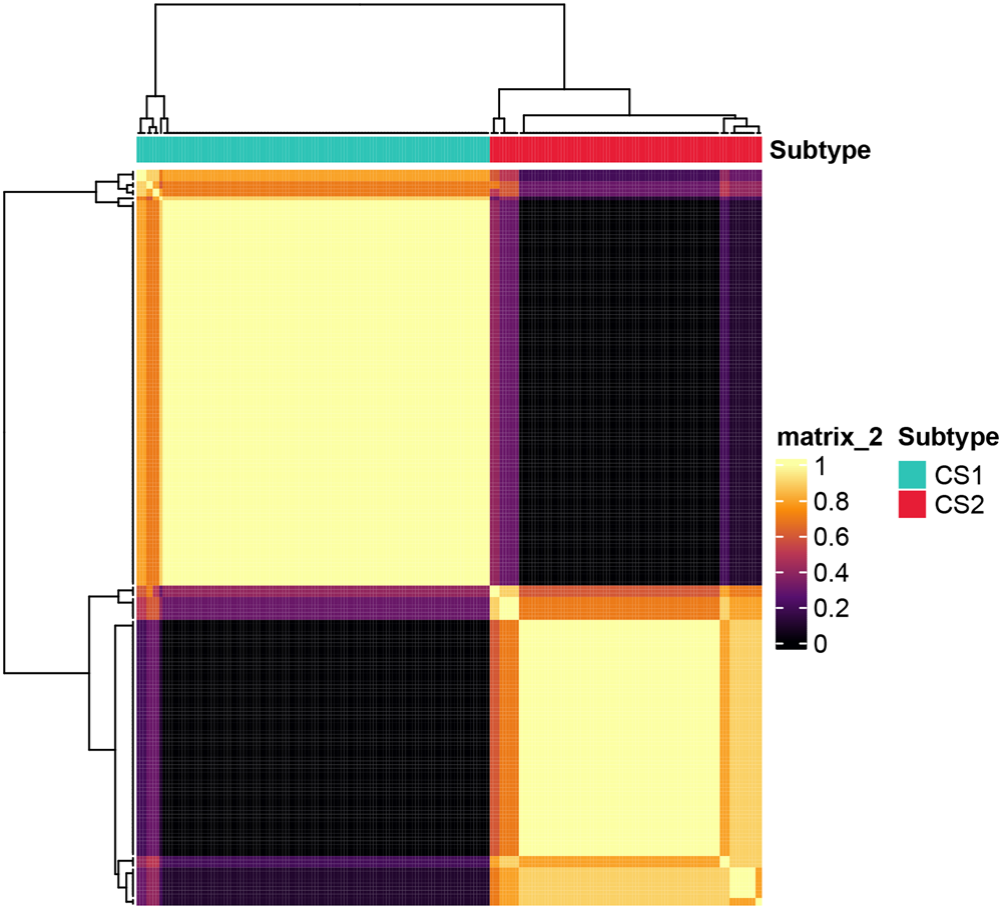
This figure incorporates the clustering consistency matrix as a heatmap, depicting the optimal clustering solution for our cohort with k = 2 clusters, derived from the integration of results across 10 advanced multi-omics clustering analysis methods. Both the x- and y-axes represent the patient samples, and each matrix element, quantifies the frequency with different patients are placed within the same cluster across the various analyses. The dendrogram positioned above the heatmap demonstrates the hierarchical clustering order of patient samples, effectively separating them into two distinct clusters.

**Fig. 3:**
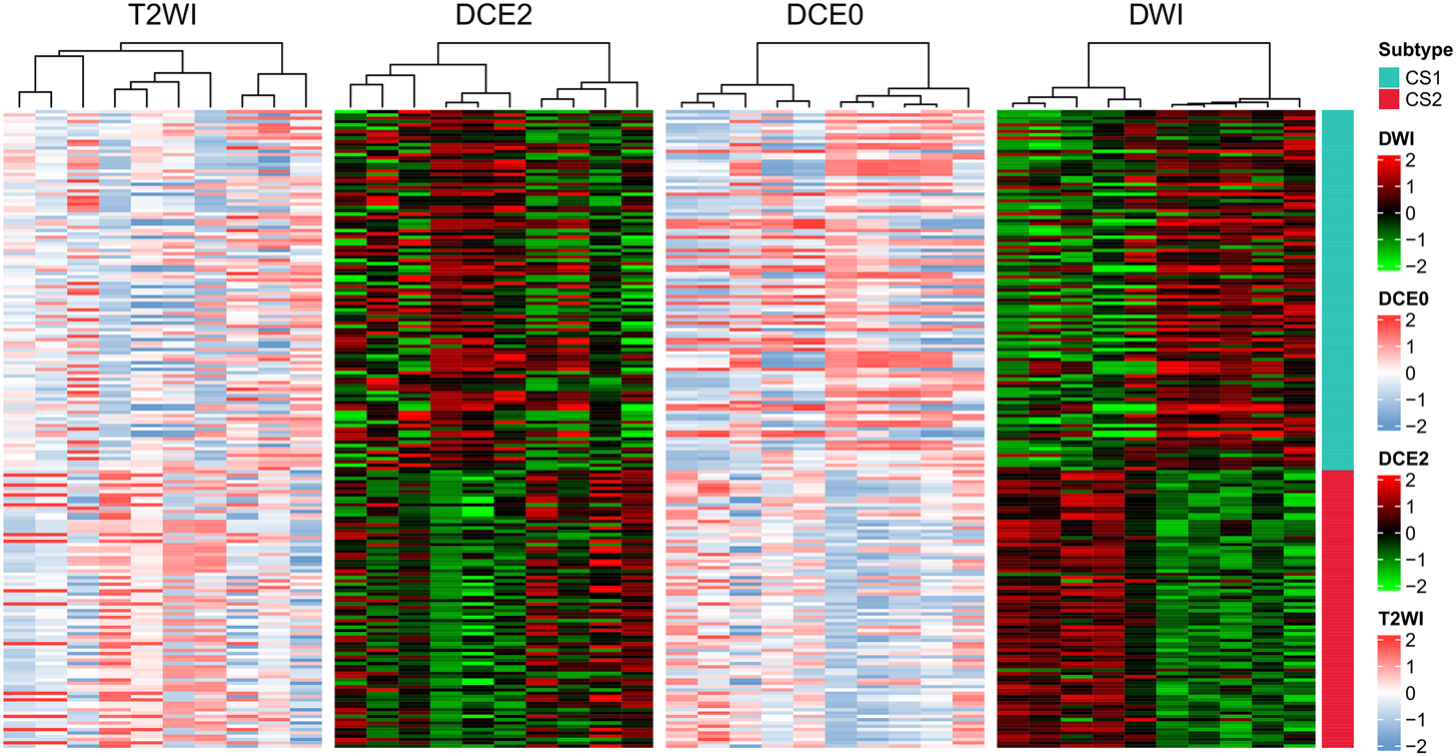
A heatmap of the distribution patterns of radiomic features in two different clusters. It clearly shows that the radiomic features vary between the different clusters.

The data indicates that the CPI reaches its peak at k = 2, suggesting that a classification count of 2 is the most optimal for this analysis (Fig. 1). Setting k = 2, ten advanced multi-omics clustering analysis methods are employed for clustering. A consistency analysis of these ten results yields a clustering consistency matrix (Fig. 2) and a heatmap of the distribution patterns of radiomic features in two different clusters. It clearly shows that the radiomic features vary between the different clusters (Fig. 3).

### Association between clustering analysis and clinicopathological characteristics

The mean age of patients in Cluster 1 (*n* = 109) was 55.3 ± 10.6 years, and in Cluster 2 (*n* = 84), it was 54.4 ± 11.0 years (*p* = 0.57) (Table 2). It was observed that the distribution of molecular subtypes across both clusters showed a significant difference (*p* = 0.03). In our study, the distribution of the molecular subtype Luminal A differed notably between the two clusters, with 16.5% (18/109) in Cluster 1 and 29.8% (25/84) in Cluster 2. This pattern is indicative of a significant variation in hormone receptor status across the clusters. The differences were particularly pronounced in both ER and PR. A statistically significant difference was found in ER status between the clusters (*p* = 0.01). Similarly, PR status also showed a significant variance (p = 0.04) (Table 2). Significant differences were observed in maximum and volume between two clusters. Cluster 1 tumors were larger, averaging 3.2 ± 1.7 cm, compared to Cluster 2, which averaged 1.6 ± 0.6 cm (*p* < 0.01). Additionally, the average volume was substantially greater in Cluster 1, at 7032.3 ± 5892.4 mm^3^, versus 1319.5 ± 880.4 mm^3^ in Cluster 2 (*p* < 0.01). Histological grades also varied significantly between the clusters (*p* < 0.01), suggesting differences in tumor aggressiveness or stage (Table 2). A significant difference was found in LNM (*p* = 0.01), but not in LVI (*p* = 0.16). No significant differences were observed in parameters such as Around, FGT, Location, BEP, Type, TIC, T2WI signals, and DWI signals, except for Edema, which showed a significant difference (*p* < 0.01) (Table 2).

**Table 2:** Clinical, Histologic, and Radiological characteristics of patients by Clustering

Characteristic	Level	Cluster = 1 (*n* = 109)	Cluster = 2 (*n* = 84)	*p* value
Age (years)*	Mean ± SD	55.3 ± 10.6	54.4 ± 11.0	0.57
Molecular subtypes	Luminal A	18 (16.5)	25 (29.8)	**0.03**
	Luminal B	50 (45.9)	42 (50.0)	
	HER2-positive	13 (11.9)	5 (6.0)	
	Basal-like	28 (25.7)	12 (14.3)	
ER	Negative	40 (36.7)	16 (19.0)	**0.01**
	Positive	69 (63.3)	68 (81.0)	
PR	Negative	63 (57.8)	35 (41.7)	**0.04**
	Positive	46 (42.2)	49 (58.3)	
HER2	Negative	44 (40.4)	36 (42.9)	1
	Positive	65 (59.6)	48 (57.1)	
Ki67	<14%	34 (31.2)	35 (41.7)	0.18
	≥14%	75 (68.8)	49 (58.3)	
Maximum(cm)	Mean ± SD	3.2 ± 1.7	1.6 ± 0.6	**<0.01**
Volume(mm^3^)	Mean ± SD	7032.3 ± 5892.4	1319.6 ± 880.4	**<0.01**
Histological grades	I	18 (16.5)	37 (44.0)	**<0.01**
	II	78 (71.6)	39 (46.4)	
	III	13 (11.9)	8 (9.5)	
LNM	Negative	55 (50.5)	58 (69.0)	**0.01**
	Positive	54 (49.5)	26 (31.0)	
LVI	Negative	76 (69.7)	67 (79.8)	0.16
	Positive	33 (30.3)	17 (20.2)	
Around	Left	49 (45.0)	44 (52.4)	0.38
	Right	60 (55.0)	40 (47.6)	
FGT	a	3 (2.8)	6 (7.1)	0.27
	b	34 (31.2)	26 (31.0)	
	c	61 (56.0)	39 (46.4)	
	d	11 (10.1)	13 (15.5)	
Location	Superior external	43 (39.5)	44 (52.4)	**0.02**
	Inferior external	16 (14.7)	12 (14.3)	
	Superior internal	21 (19.3)	19 (22.6)	
	Inferior internal	10 (9.2)	6 (7.1)	
	Posterior areola	14 (12.8)	0 (0.0)	
	Central region	5 (4.6)	3 (3.6)	
BEP	Minimal	39 (35.8)	33 (39.3)	0.13
	Mild	52 (47.7)	46 (54.8)	
	Moderate	15 (13.8)	5 (6.0)	
	Significant	3 (2.8)	0 (0.0)	
Type	Non-mass	10 (9.2)	7 (8.3)	1
	Mass	99 (90.8)	77 (91.7)	
TIC	I	3 (2.8)	4 (4.8)	0.07
	II	35 (32.1)	39 (46.4)	
	III	71 (65.1)	41 (48.8)	
Edema	No	33 (30.3)	62 (73.8)	**<0.01**
	Yes	76 (69.7)	22 (26.2)	
T2WI signals	Low	24 (22.0)	26 (31.0)	0.28
	Equal	45 (41.3)	27 (32.1)	
	High	40 (36.7)	31 (36.9)	
DWI signals	Low	1 (0.9)	0 (0.0)	0.51
	Equal	6 (5.5)	7 (8.3)	
	High	102 (93.6)	77 (91.7)	

The bold values represent statistically significant results.

## Discussion

Clustering primarily serves as an analytical technique that extracts meaningful information based on the pattern characteristics of a target data set. It divides unlabelled data into different clusters, where the similarity within each cluster is high, and the dissimilarity between clusters is significant. Currently, this approach is applied in many fields^10^. Cluster analysis aims to categorize data points within a population into Clusters where members of each cluster share more similarities with each other than with those in different clusters^9^. We employed unsupervised cluster analysis to uncover natural groupings within the imaging features we extracted, leading to the identification of intrinsic phenotypes characterized by distinct and measurable imaging patterns. This method reflects the clustering techniques pivotal in uncovering the inherent molecular subtypes of breast cancer. To visualize and interpret these imaging phenotypes, we generated heat maps akin to microarray expression clustering dendrograms. This visualization aids in beginning to understand the correlation between the imaging presentation of cancer and its molecular profile, potentially offering insights into the likelihood of cancer recurrence.

Lee et al demonstrated the potential of machine learning in radiomics for predicting prognostic biomarkers and molecular subtypes of breast cancer, emphasizing the role of tumor heterogeneity and angiogenesis properties on MRI^6^. Romeo et al applied a machine learning-based radiomics approach to hybrid 18F-FDG PET/MRI for predicting axillary lymph node involvement in breast cancer^11^. Szep et al demonstrated that whole-tumor ADC texture analysis could predict hormonal status in breast cancer masses^12^. Furthermore, Araz et al explored the role of preoperative 18F-FDG PET/CT radiomics features in predicting hormone receptor positivity in primary breast tumors^13^. While their study found limited predictive value in radiomics parameters for hormone receptor status, it highlights the complexity and challenges in correlating radiomics features with specific tumor characteristics. These findings support our findings on the utility of radiomics in assessing tumor aggressiveness and invasion potential. Unlike previous research primarily reliant on qualitative interpretation for identifying distinct imaging patterns linked to histopathologic correlates^14^, our study introduces a systematic, quantitative evaluation framework. Our study adopts a hierarchical clustering method, providing a structured approach to discern intrinsic imaging phenotypes of breast tumors. The study’s findings reveal statistical significance in molecular subtypes, ER, PR, maximum, volume, histological grading, edema, LNM, and location between the two clusters. The significant variations observed in these areas underscore the efficacy of radiomics clustering analysis in distinguishing breast cancer cases based on their intrinsic phenotypes. This differentiation is crucial, as it paves the way for more personalized treatment approaches, tailoring strategies to the specific characteristics and needs of each tumor type. The ability to categorize breast cancer more precisely based on these key parameters holds significant potential for enhancing the effectiveness of treatment plans and improving patient outcomes.

In our cluster analysis, Cluster 2 demonstrated more favorable prognostic features compared to Cluster 1. Specifically, Cluster 2 exhibited higher ER and PR positivity rates, a larger proportion of the Luminal A subtype, and notably smaller tumor sizes. Additionally, tumors in Cluster 2 were more likely to be classified as histological grade I. The lower incidence of LNM and peritumoral edema in Cluster 2 further underscores its better prognosis. These statistically significant differences between the clusters highlight distinct biological behaviors and potential treatment responses, with Cluster 2 presenting characteristics that are generally associated with a more favorable outcome in breast cancer management, align with recent advancements in breast cancer research. Molecular subtypes, particularly those defined by hormone receptor status, have been widely recognized for their prognostic implications. ER-positive and PR-positive tumors often suggest a better prognosis and responsiveness to hormonal therapies, which could lead to improved survival rates^15^. The luminal A subtype of breast cancer is known for its favorable prognosis, attributed to distinct biological traits and effective therapy responses. Characterized by high expression of ER and PR receptors, luminal A tumors are particularly receptive to hormone therapy, enhancing patient outcomes. Additionally, these tumors exhibit a lower Ki67 index, reflecting reduced cell proliferation. This slower growth pace leads to a less aggressive disease trajectory, providing a broader window for successful treatment interventions and hindering progression to more advanced stages^16,17^. Conversely, tumors with negative hormone receptor status, particularly those falling into the triple-negative category, are often associated with a more aggressive course and poorer outcomes^18^. In our study, we acknowledge the unique composition of our cohort, characterized by an underrepresentation of the Luminal A subtype, a divergence from the expected distribution observed in broader breast cancer populations^19^. The distribution of proportions in this study closely mirrored those reported by Hashmi et al.^17^, featuring a notably higher percentage of the Luminal B subtype. This variability in subtype prevalence, influenced by factors such as geography, ethnicity, genetic, and Microbiota^20^. Xu et al investigated molecular phenotypes and clinical characteristics of familial hereditary breast cancer. The study results showed significant differencesbetween hereditary and non-hereditary breast cancers, including histological grading (grade II/III), LNM, PR, and HER2^21^. Their findings resonate with our study, underscoring the complexity and diversity of breast cancer subtypes. Despite this, the strength of our methodological approach, particularly the use of unsupervised learning for cluster analysis, allows us to mitigate potential biases and ensure the identification of meaningful radiomic patterns that are intrinsic to the data.

Tumor size, histological grading, and tumor location are also critical in determining the aggressiveness of the disease. Larger tumors and those with higher histological grades are generally indicative of a more advanced disease state, potentially leading to lower survival rates. Literature indicates that smaller breast cancers (<2 cm) are associated with higher survival rates post-surgery, including after breast-conserving surgery, as reflected in longer overall survival (OS) and breast cancer cause-specific survival (BCSS)^16,22^. This trend may be attributed to various factors inherent to the biological and pathological nature of larger tumors. For instance, larger tumors, due to their extended growth period, are more prone to accumulating genetic mutations which can escalate cancer aggressiveness and resistance to treatment. Additionally, their capacity to induce angiogenesis enhances both tumor nourishment and metastatic potential. The propensity of larger tumors to invade adjacent tissues and lymph nodes marks a pivotal step towards metastasis. Such tumors also experience alterations in hormone receptor status and growth factor receptor expression as they grow. Moreover, the heterogeneity within larger tumors, characterized by a wider variety of cancer cell types, complicates treatment due to the varied response among these cell populations. Recognizing the importance of tumor size, the AJCC 8th edition has incorporated it as a critical parameter in staging breast cancer, demarcating T1 and T2 stages at the 2 cm threshold, underscoring the significance of tumor size not only in prognostication but also in guiding therapeutic decisions^23^. Edema, while not as extensively studied as other factors, could provide additional insights into the tumor microenvironment and its interaction with the surrounding tissues, which might have implications for disease progression and metastatic potential. In the study by Zeyan Xu et al., edema is a promising predictive factor for lymph node metastasis in breast cancer, which is very important for patients with early-stage breast cancer and may help in formulating treatment plans. Additionally, a positive correlation was observed between BES and various aggressive clinicopathological factors (*p* < 0.05)^24^.

Similarly, the presence of lymph node metastasis is a well-established marker of poor prognosis, as it signifies the spread of cancer cells beyond the primary tumor site. Lymph node metastasis in breast cancer patients is a critical factor that significantly influences prognosis. The presence of cancer cells in the lymph nodes, particularly the axillary lymph nodes, is often considered a hallmark of the spread of the disease beyond the primary tumor site. This metastatic spread is a key determinant in staging breast cancer, which in turn guides treatment decisions and helps predict patient outcomes. Patients with lymph node involvement generally have a poorer prognosis compared to those without. Recent studies, including the development of nomogram models, have highlighted several independent predictive factors for axillary lymph node metastasis in breast cancer patients, and these factors include tumor size, primary site, molecular subtype, and histological grading^25^. Lyu et al analyzed factors influencing sentinel lymph node metastasis in breast cancer, highlighting the importance of molecular subtypes, tumor size, and histological grade in predicting lymph node involvement^26^. Wang et al analyzed risk factors of axillary lymph node metastasis and prognosis in T1 breast cancer using the SEER database, highlighting the impact of tumor size, histological grade, and subtype on lymph node involvement and survival outcomes^27^. Several studies suggest that the location of a breast tumor influences disease-free survival (DFS)^28^, with tumors situated in the inner/lower quadrants being anatomically closer to the internal mammary chain, thus having a higher propensity for metastasis. Our current study provides further insights. Specifically, Cluster 1 tumors were predominantly found in the inner and lower quadrants, areas known for their closer anatomical proximity to the internal mammary chain and, consequently, a higher risk of metastasis. In contrast, Cluster 2 tumors were more frequently located in the outer and upper quadrants, regions typically associated with a lower metastatic risk. Notably, all cases of tumors behind the areola were grouped in Cluster 1. This distribution aligns with the hypothesis that Cluster 1 may have a less favorable prognosis compared to Cluster 2, underscoring the critical role of anatomical factors in influencing breast cancer outcomes. This further validates our study’s emphasis on these factors in breast cancer assessment. Our study contributes significantly to breast cancer research by providing a comprehensive cluster analysis that correlates imaging features with key clinical and pathological parameters. We offer a novel approach to breast cancer classification, integrating imaging phenotypes with molecular typing, ER, PR, tumor size, histological grading, edema, and lymph node metastasis, these may have varying influences on the prognosis and survival outcomes in breast cancer patients. This multidimensional analysis enhances the understanding of breast cancer heterogeneity and supports the development of personalized treatment strategies. In future studies, it would be crucial to integrate these factors into survival analysis models to comprehensively understand their collective impact on patient outcomes. Such analyses could not only validate the clinical significance of our cluster analysis results but also contribute to the development of more tailored and effective treatment strategies, ultimately improving patient survival and quality of life in breast cancer.

In our analysis, several variables, including age, HER2, Ki67, LVI, Around, FGT, BEP, Type, TIC, T2WI signals, and DWI signals, did not show statistically significant differences. The absence of significant findings in these areas, while initially surprising, offers valuable insights into the complex biology of breast cancer. HER2 and Ki67 are important biomarkers in breast cancer^29^, but they did not show significant differences in two clusters in our study. This may be due to the large inter-individual variability in the expression levels of these markers in the samples or the weak correlation with the imaging features of interest in our clustering method. LVI is strongly associated with increased peritumoral lymphovascular density and more aggressive neovascularization, leading to variations in the volume and flow of blood within the tumor microcirculatory environment^30^. The radiomics model developed by Zhang et al., utilizing multiparametric MRI, effectively predicts LVI presence, demonstrating significantly shorter DFS for patients at high risk of LVI compared to those at low risk^31^. This indicates that LVI is linked to a worse prognosis in breast cancer. Studies like those conducted by Lai et al. have shown no significant difference in overall survival (OS) between patients with and without LVI^32^. Analyzing these differences may be related to variations in tumor grading and typing. Furthermore, research indicates that the presence of vascular invasion, but not lymphatic invasion, could be an indicator of high biological aggressiveness and may serve as a valid prognostic factor for breast cancer^32^. Our study did not distinguish between vascular invasion and lymphatic invasion within LVI. In our analysis, the cluster analysis results indicated no statistical significance in the Ki67 index. This finding may be partially attributed to the complexities surrounding the interpretation and application of Ki67 as a biomarker. The Ki67 index, which quantifies the proportion of proliferating genes/cells, lacks universally accepted cutoff values. While the St. Gallen International Expert Consensus has proposed a potential threshold of < 14% for Ki67^33^, Prat et al. have suggested a more stringent cutoff of > 20%^34^. This discrepancy in recommended values reflects the broader challenge of applying a uniform standard to a marker characterized by significant heterogeneity. The inherent variability in the genetic and morphological characteristics of breast tumors further complicates the establishment of a definitive Ki67 threshold. Thus, the absence of statistical significance in Ki67 in our study could be a manifestation of these underlying complexities, underscoring the need for a nuanced approach to interpreting Ki67 levels within the context of breast cancer heterogeneity. The non-significant findings for variables such as T2WI and DWI signals may indicate the inherent heterogeneity of breast cancer, suggesting that these imaging characteristics alone may not sufficiently capture the diversity of tumor biology across different subtypes.

Our study makes significant contributions to the field of breast cancer research by integrating radiomics clustering analysis with traditional histopathological markers. We provide a comprehensive approach to breast cancer classification, enhancing the understanding of tumor heterogeneity and its implications for personalized medicine. Our findings contribute to the growing body of evidence supporting the use of radiomics in breast cancer diagnosis and treatment planning. In evaluating conflicting explanations of our results, we defend our approach by highlighting the robustness of our methodology and the consistency of our findings with current literature. While some studies suggest limited predictive value of radiomics in certain aspects of breast cancer^23^, our study demonstrates its utility in differentiating intrinsic phenotypes, supporting its role in personalized treatment strategies.

While our study provides valuable insights into the classification and treatment of breast cancer using radiomics, it is important to acknowledge its limitations. Firstly, the limited sample size of 194 patients, although substantial, may not fully capture the diversity and complexity of breast cancer phenotypes. Another significant limitation is the absence of survival analysis. Our study focused on the classification of breast cancer based on radiomics and traditional histopathological markers, without delving into the long-term outcomes of these patients. Future studies with larger cohorts are necessary to validate and extend our results, and include survival data to provide a more comprehensive understanding of the clinical significance of radiomics-based classification. Additionally, our study did not incorporate genetic testing results, which could provide deeper insights into the molecular underpinnings of the observed phenotypes. The lack of genetic information represents a missed opportunity to explore the interplay between genotypic and phenotypic characteristics of breast cancer. In conclusion, while our study makes significant contributions to the field of breast cancer research, addressing these limitations in future studies will be crucial for advancing our understanding of breast cancer and improving patient care. Expanding the sample size, including survival analysis, and integrating genetic testing results are key areas for future research that could significantly enhance the impact and applicability of our findings.

## Methods

### Study Sample

**Fig. 4:**
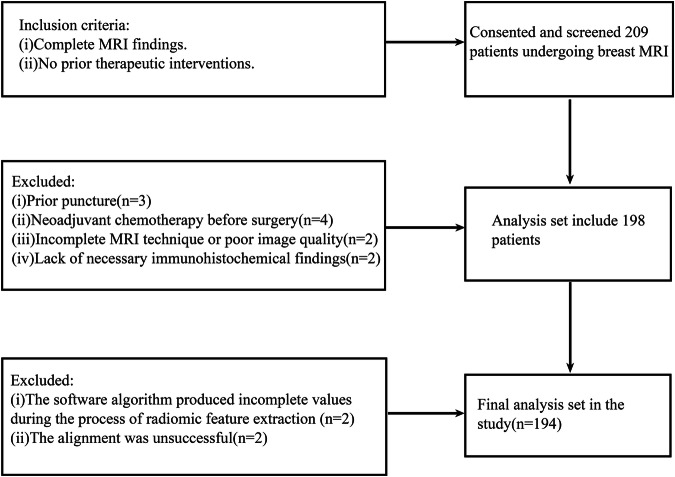
Patient inclusion and exclusion flowchart. This flowchart shows the inclusion and exclusion situations for the study participants.

The study retrospectively analyzed breast cancer cases from MA’ANSHAN People’s Hospital, spanning from September 2019 to October 2023. Inclusion Criteria: (i) Complete MRI findings. (ii) No prior therapeutic interventions. (iii) Pathological results and immunohistochemical data within a week following the MRI examination. In this study, several exclusion criteria were applied. These included patients who had undergone prior puncture or radiotherapy (*n* = 3), those who received neoadjuvant chemotherapy before surgery (*n* = 4), and cases with incomplete MRI technique or poor image quality (*n* = 2). Additionally, patients were excluded if they lacked necessary immunohistochemical findings (*n* = 2), if the software algorithm produced incomplete values during the radiomic feature extraction process (*n* = 2) or The alignment was unsuccessful (*n* = 2). (Fig. 4). Ethical Considerations: The study was conducted in accordance with the ethical standards of both the institutional and national research committees, including the Declaration of Helsinki. It was approved by the Ethics Committee of MA’ANSHAN People’s Hospital (approval number: 2022007005) and the requirement for written informed consent was waived.

### MRI image acquisition

Imaging was performed using the German Siemens Prisma 3.0 T superconducting magnetic resonance scanner with an 18-channel phased array breast-specific coil. Patients were positioned prone, feet first. The imaging techniques included T2-weighted imaging (T2WI), ZOOMit-Diffusion weighted imaging (ZOOMit-DWI), and Dynamic Contrast-Enhanced MRI (DCE-MRI) phases 0 and 2.

The specifics of the sequence parameters utilized in this analysis are provided in Table 3. DCE-MRI Sequence consists of nine phases, and the contrast agent (Omniscan, GE Pharmaceuticals) was administered at the end of the first phase, using a high-pressure injector. The contrast agent dosage was 0.1 mmol/kg of body weight, with a flow rate of 2.5 mL/s, followed by a 20 mL saline flush.

**Table 3:** Imaging parameters of breast MRI

Parameter	T1WI	T2WI	DWI	DCE-MRI
Sequence Type	TSE	TSE	ZOOMit-DWI	Fast Gradient-Echo
Field of View (mm)	340×340	340×340	340×340	360×360
Matrix	384×286	384×286	190×190	384×286
TR (ms)	5.3	6000	6000	4.13
TE (ms)	2.46	63	63	1.3
Slice Thickness(mm)	1.5	4	4	1.2
Interval (mm)	Flash-3D	0.8	0.8	Flash-3D
b-value (s/mm²)	-	-	50, 500, 1000,1500	-
Contrast Agent (DCE-MRI only)	-	-	-	Omniscan (GE Pharmaceuticals)
Injection Rate (mL/s)	-	-	-	2.5
Phases (Scanning and Enhancement)	-	-	-	1 + 8 phases
Scanning Time per Phase (s)	-	-	-	60 s

*TSE* Turbo Spin Echo, *TR* Repetition Time, *TE* Echo Time.

### Image processing

The DWI image was based on a b-value of 1500 s/mm². This parameter was chosen as it is typically optimal for highlighting differences in tissue diffusivity, which is particularly relevant in the context of breast tumor characterization^4^.

For the assessment of tumor morphology, all MRI images underwent a thorough review by two experienced breast radiologists, who had 10 and 23 years of experience, respectively, in the diagnostic imaging of breast diseases. This review was conducted while the radiologists were blinded to the patients’ clinical history. The evaluation adhered to the criteria set forth in the 2013 Breast Imaging Reporting and Data System (BI-RADS) MR lexicon^35^. In cases where patients presented with multiple lesions, the largest lesion was selected for detailed evaluation. The radiologists recorded several key aspects of each lesion, including Location and Type (mass or non-mass), morphology (round, round-like, or irregular), Margin (clear or indistinct), Maximum Diameter (measuring the largest dimension of the lesion), Fibroglandular Tissue (FGT) and Background Parenchymal Enhancement (BPE). Additionally, Signal Intensity on T2WI and DWI (slightly low, equal, or high signal) and edema in Adjacent Breast Parenchyma.

The presence of intratumoral high signal intensity (SI) was meticulously assessed on T2WI and DWI sequences, and peritumoral edema was meticulously assessed on T2WI^36,37^. Intratumoral high SI was visually defined when the lesion’s SI was stronger than that of vessels, water, or the surrounding parenchymal tissue, and peritumoral edema was determined based on the observation of high SI around the tumor on T2WI^36,37^.

### Tumor segmentation and registration

**Fig. 5:**
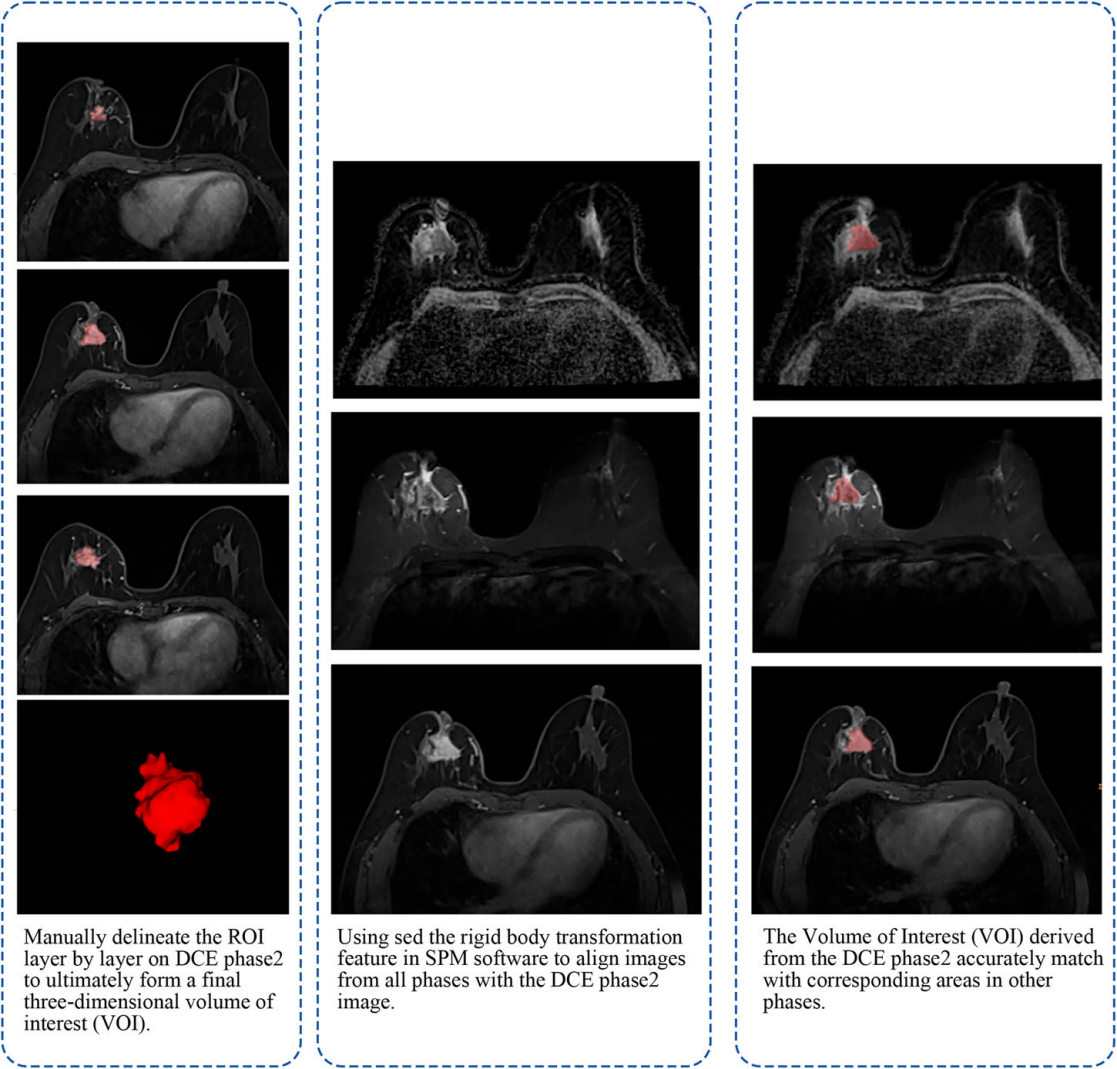
Lesion segmentation and image processing procedure. Manually delineate the VOI on DCE phase 2, align images using SPM software, and match the VOI with corresponding areas in other phases.

The tumor boundary was delineated by two experienced radiologists (Radiologist 1, Q.X. 10 years in breast cancer diagnosis. Radiologist 2, H.Y.S. 23 years in breast cancer diagnosis.) using ITK-SNAP software (http://www.itk-snap.org). Q.X. carefully segmented the tumor on the enhanced phase 2 images, including cystic and necrotic areas. H.Y.S. checked the segmentation, if they had different opinions, they discussed to reach a consensus. After segmentation, a three-dimensional Volume of Interest (VOI) were obtained (Fig. 5). Another two radiologists (Radiologist 3, W.W.L. 9 years in breast cancer diagnosis. Radiologist 4, Y.H.K. 22 years in breast cancer diagnosis.) employed the same segmentation method to randomly re-segment 40 patient cases. Initially, W.W.L. conducted the preliminary segmentation, after which Y.H.K. reviewed the work. In instances where discrepancies arose, the two radiologists engaged in a discussion to reach a consensus. To quantitatively assess the consistency of our segmentation, we utilized the DICE coefficient, a statistical tool that measures the overlap between two segmentations. Additionally, we evaluated the consistency of extracted radiomic features using the Intraclass Correlation Coefficient (ICC) provided in Supplementary Fig. 1.

The segmentation method we use is the semi-automatic outlining method in ITK-SNAP, which belongs to one kind of Active Contour Model, and the specific operation steps are as follows:

(i) Data Importation: Importing the breast enhancement data from the DCE-MRI Phase 2 images into ITK-SNAP software.
(ii) Initial Thresholding: We utilized the “Segment 3D” tool within ITK-SNAP to apply an initial thresholding technique. We carefully adjusted the lower and upper threshold levels to accurately capture the tumor boundaries while minimizing the inclusion of non-tumor tissues.
(iii) Bubble Tool Activation: After setting the appropriate thresholds, we used the “Bubble Tool” at the cursor’s position to begin the rendering process.
(iv) Manual Adjustment: Recognizing the limitations of automated processes in accurately capturing complex tumor geometries, we proceeded to manually adjust the segmented tumor in all three anatomical directions—axial, sagittal, and coronal planes, ensuring that the final segmentation included all tumor regions while excluding adjacent non-tumorous tissue.

To ensure consistency across the different modalities, we used the “Registration” module in SPM software to align images to the second phase image. This alignment was crucial for accurately matching the VOI derived from the second phase with corresponding areas in other modalities. After alignment, the VOI and images were imported into ITK-SNAP for a thorough visual inspection. Any discrepancies identified led to manual adjustments of the VOI, ensuring precise and uniform tumor segmentation across the different MRI sequences.

### Radiomics feature extraction and consensus clustering

Radiomics feature extraction for each sequence of mpMRI was conducted using the open-source Python package ‘pyradiomics’ (version 3.0.1, https://pyradiomics.readthedocs.io/en/latest/), an IBSI-compliant tool. The feature extraction included two steps, image preprocessing and feature calculation. The preprocessing of images included resampling image to an isotropic voxel size of 3 mm and grayscale discretization with a bin width of 10. Additionally, image intensity normalization was applied. In total, 1158 radiomics features were extracted for each modalities, encompassing shape, first-order, and second-order texture features, as well as high-order features derived from wavelet-transformed and Laplace of Gaussian transformed images.

In our study, we employed unsupervised clustering analysis to categorize patients using the multi-parametric’s radiomics features, including T2WI, DWI, DCEphase0, and DCEphase2. This comprehensive clustering analysis was conducted on the entire cohort of 194 patients, ensuring a robust and thorough exploration of the radiomics data and its potential clinical correlations. The process involved several key steps: (i) Feature Dimension Reduction: Initially, we applied the Uniform Manifold Approximation and Projection (UMAP) method for feature dimension reduction^38^. This step was crucial in simplifying the complex radiomics data while retaining essential information.For each imaging modality, we preserved 10 key features, ensuring a comprehensive yet manageable dataset for subsequent analysis (Supplementary Fig. 2). (ii) Determination of the optimal number of clusters: The cluster prediction index (CPI) was used to identify the optimal number of clusters (k). CPI is termed as the Cluster Prediction Index, the maximum CPI corresponding to the optimized ‘k’. (iii) Integrative clustering: For our data was multi-modalities, similar to multi-omics data, so we applied the advanced multi-omics clustering algorithms from the MOVICS package to perform multi-modalities clustering analysis^39^. As introduced in the Lu et al.’s paper^39^, 10 advanced multi-omics clustering analysis methods were performed, then all the clustering results from 10 algorithms are in hand, MOVICS calculates a matrix ${M}_{n\times n}^{\left(t\right)}$ per algorithm where $n$ is the number of patients, and t represents the algorithm t ($2\le t\le 10$), and ${M}_{{ij}}^{\left(t\right)}$ only when the patient $i$ and $j$ are clustered in the same subtype, otherwise ${M}_{{ij}}^{(t)}=0$. After get all results from 10 algorithms, MOVICS calculates a consensus matrix ${CM}=\mathop{\sum }\nolimits_{t=1}^{{t}_{\max }}{M}^{(t)}$, and$\,{{cm}}_{{ij}}\in [\mathrm{0,10}]$. Such matrix represents a robust pairwise similarities for samples because it considers different multi-omics integrative clustering algorithms. MOVICS conducted a hierarchical clustering to identify a stable clustering result. (iv) Comparison of Radiomics features in different clusters: Once the clusters were established, the radiomics feature distribution heatmap was given to show whether the features’ distributions were different between different cluster.

### Histological evaluation

Lymph node metastasis (LNM) occurrence, lymphovascular invasion (LVI) status, and pathological type were recorded post surgery. LVI was positive when cancer cells were found in lymphatic vessels or small blood vessels outside the tumor. Streptavidin-peroxidase immunohistochemistry was used to determine the expression statuses of estrogen receptor (ER), progesterone receptor (PR), HER2, and Ki67 in surgically excised breast cancer tissues. Molecular subtypes were categorized based on these markers: Luminal A (ER/PR ≥ 1%, HER2-negative, Ki67 < 14%), Luminal B (ER/PR ≥ 1% with either Ki67 ≥ 14% or HER2-positive), Basal-like (ER < 1%, PR < 1%, HER2-negative), and HER2-positive (ER < 1%, PR < 1%, HER2-positive). Ki67 expression was categorized as low (<14%) or high (≥14%). Histologic grade, determined using the Nottingham grading system, was categorized as low malignancy (grade I, score 3–5), moderately malignant (grade II, score 6–7), and highly malignant (grade III, score 8–9), with the World Health Organization adopting this system as the standard for invasive breast cancer. ER or PR positivity was defined as positive nuclear staining in more than 10% of cells, while HER2 positivity was indicated by a CerbB-2 value of 3+ or a fluorescence in situ hybridization (FISH) amplification ratio≥2.0.

### Statistical analysis

All statistical analyses were conducted using R software (version 4.2.1, www.r-project.org). Continuous variables were described as mean ± standard (SD) deviation when normally distributed, and as median with interquartile range (IQR) for non-normal distributions. To compare clinical characteristics across the final clusters, categorical data were analyzed using the Fisher’s exact test or the chi-square (χ^2^) test, while continuous data were evaluated using the analysis of variance (ANOVA) test or Kruskal-Wallis test, accroding to the data distribution. All tests were two-sided, with a P-value of less than 0.05 considered statistically significant.

### Supplementary information

Supplementary Figure 1 and Supplementary Figure 2

## Data Availability

Data generated in this study are available upon reasonable request from the corresponding author.

## Code availability

The code used for statistical calculations in this analysis is publicly available in https://github.com/yzhizai/radiomics_phenotype.git.
