# Supplementary material for: Integrative radiomics clustering analysis to decipher breast cancer heterogeneity and prognostic indicators through multiparametric MRI
Source: NPJ Breast Cancer. 2024 Aug 7;10:72. doi: 10.1038/s41523-024-00678-8 (PMC11306571; doi:10.1038/s41523-024-00678-8)
Supplement: Supplementary file 1 — Supplementary Figure 1 and Supplementary Figure 2 [file 41523_2024_678_MOESM1_ESM.pdf]

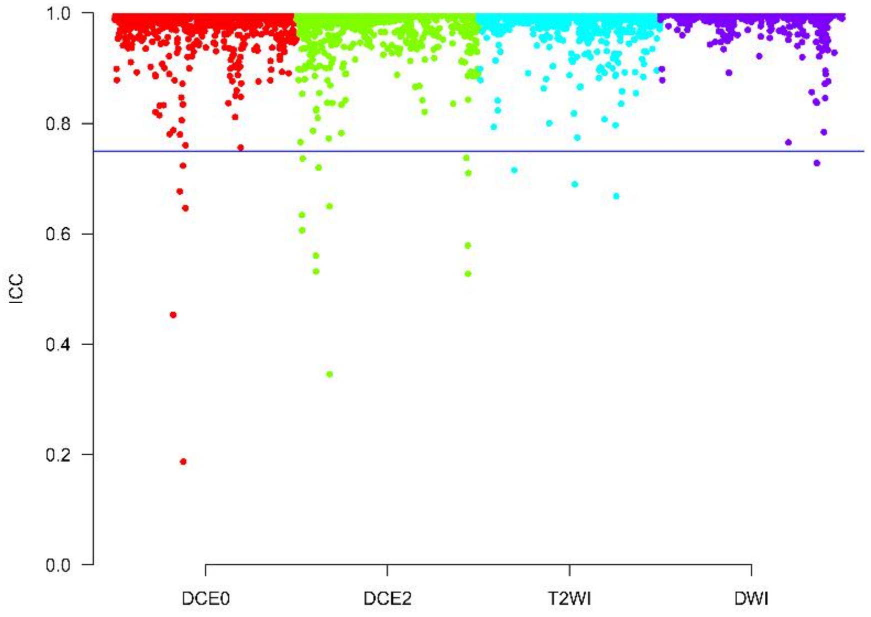

Supplementary Figure 1: Manhattan figure. Forty randomly selected patient images underwent re-segmentation by two additional radiologists utilizing the same segmentation approach. The DICE coefficient yielded a value of 0.968±0.035. The ICC values for DCE0, DCE2, T2WI, and DWI are 0.980±0.044, 0.978±0.050, 0.984±0.029, and 0.992±0.020, respectively.

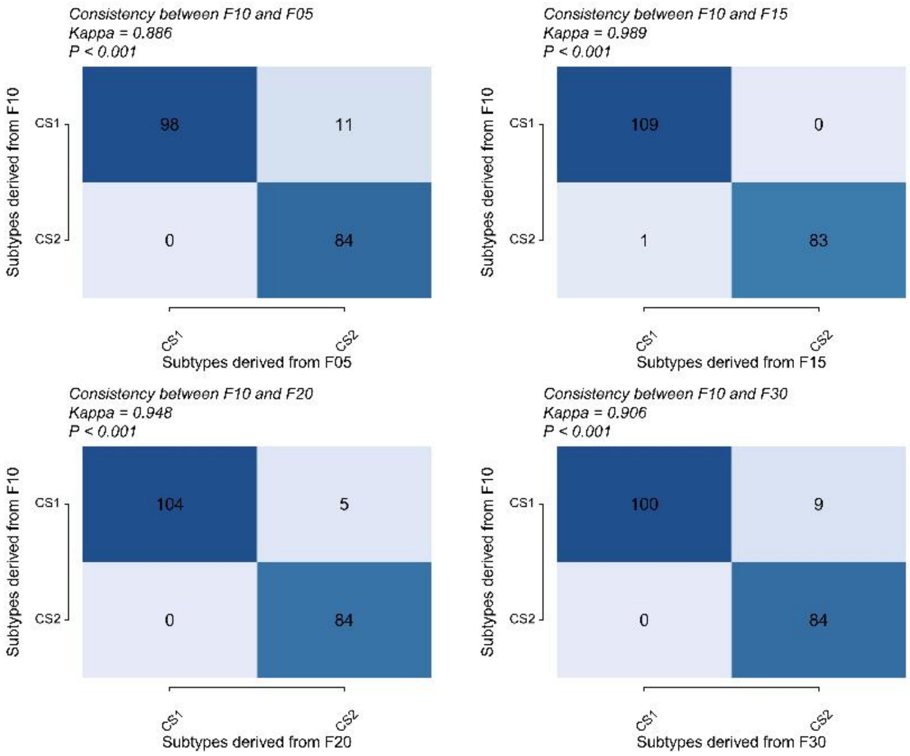

Supplementary Figure 2: This figure illustrates the results of our experimentation with varying numbers of features—specifically, 5, 10, 15, 20, and 30—and the subsequent assessment of consistency using the kappa statistic for each feature set. The kappa values, represented in the graph, all surpass the 0.8 mark, with several nearing the perfect consistency score of 1. This graphical representation underscores the minimal impact of the number of features on the clustering outcomes, highlighting the UMAP method's efficiency in preserving the topological structure of the data throughout the dimensionality reduction process. The consistent high kappa scores across different feature sets emphasize the robustness of our analysis technique, regardless of the feature quantity selected.
